# Air Chamber Atmospheric Plate

**Published:** 1855-04

**Authors:** George F. Waters

**Affiliations:** Dentist, Boston.


### ARTICLE VII.

*Air Chamber Atmospheric Plate.* By GEORGE F. WATERS, Dentist, Boston.

MESSRS. EDITORS:—Permit me to communicate to you what I believe to be an improvement in the air chamber of atmospheric plates, for the support of single teeth, or of whole sets. The subject of this paper pressed itself upon my attention three years or more ago, from having quite a number of *flat*

*cases* come under my observation. In these cases such had been the loss by absorption, that the alveolar ridge offered but little or no resistance to the sliding forward of the plate. The first attempt at gaining immobility was by cutting out from the centre of the plate a piece the size of the required cavity, and then soldering on to the lingual side of the plate another piece, or the same, enlarged sufficiently to cover the cavity. Subsequently, however, I have been obliged to modify the cavity, and it is to this modification that I would call attention at this time. And first let me say, that my principal objection to the above form of cavity was found in attempting partial sets by atmospheric pressure. It *frequently* succeeded, but not *uniformly*. Often the plate could not be pressed home, so as to give the tooth, or teeth, a firm bearing, on account of the edge of the cavity next the teeth striking against the gum before it was firmly in place. To remedy this, the cavity may be modified by cutting through that part of it opposite the bearing of the teeth—which should have a square edge—and up to the approximate sides. Bending it out from the body of the plate to the lingual side, the surface may be distended by a small round-headed hammer, sufficiently to form a good sized cavity—wedge-shaped—the thin part pointing towards the teeth. Two or three of these cavities may be put into a plate, as the case may require. For whole sets, cutting through the back part of the plate, about one quarter of an inch from the edge, I run the saw across from one ridge to the other, coming up on each side to within about one eighth of an inch of the apex of the ridge. The little strip thus partially severed from the plate is now to be forced under, (both edges being finished,) for the double purpose of stiffening the plate, and forming the back square edge of the cavity. I have not deemed it important to send drawings, but will do so if you desire, or a sample in base metal. I think that in using this form of cavity in plates, there will be no difficulty in supporting dentures, in any case, by atmospheric pressure. It has worked well in all cases where I have as yet tried it.

BOSTON, 121 Court street, *Feb. 22d,* 1854.

